# Universal Bacterium-Vectored COVID-19 Vaccine Expressing Early SARS-CoV-2 Conserved Proteins Cross-Protects Against Late Variants in Hamsters

**DOI:** 10.3390/vaccines13060633

**Published:** 2025-06-12

**Authors:** Qingmei Jia, Helle Bielefeldt-Ohmann, Saša Masleša-Galić, Richard A. Bowen, Marcus A. Horwitz

**Affiliations:** 1 Division of Infectious Diseases, Department of Medicine, 32-150 Center for Health Sciences, School of Medicine, University of California—Los Angeles, 10833 Le Conte Avenue, Los Angeles, CA 90095, USA; qjia@mednet.ucla.edu (Q.J.); sgalic@mednet.ucla.edu (S.M.-G.); 2 Australian Infectious Diseases Research Centre, University of Queensland, St Lucia, QLD 4072, Australia; h.bielefeldtohmann1@uq.edu.au; 3 Department of Biomedical Sciences, Colorado State University, Fort Collins, CO 80523, USA; rbowen@colostate.edu

**Keywords:** vaccine, single-vector platform, LVS Δ*capB* vector, COVID-19, SARS-CoV-2, membrane protein, nucleocapsid protein, RdRp, Delta variant, Omicron variant

## Abstract

**Background/Objectives:** Severe acute respiratory syndrome coronavirus 2 (SARS-CoV-2), the causative agent of Coronavirus Disease 2019 (COVID-19), has rapidly evolved, giving rise to multiple Variants of Concern—including Alpha, Beta, Gamma, Delta, and Omicron—which emerged independently across different regions. Licensed COVID-19 vaccines primarily target the highly mutable spike protein, resulting in reduced efficacy due to immune escape by emerging variants. Previously, we developed a live attenuated *Francisella tularensis* LVS Δ*capB* single-vector platform COVID-19 vaccine, rLVS Δ*capB*/MN, expressing the conserved membrane (M) and nucleocapsid (N) proteins from the early SARS-CoV-2 WA-01/2020 strain. In this study, we evaluate the efficacy of rLVS Δ*capB*/MN and an enhanced version, rLVS Δ*capB*::RdRp/MN, which additionally expresses the conserved RNA-dependent RNA polymerase (RdRp) protein from the same strain, in a hamster model. **Methods:** Both vaccine candidates were administered orally or intranasally to golden Syrian hamsters (equal numbers of males and females) and evaluated against intranasal challenge with SARS-CoV-2 Delta (B.1.617.2-AY.1) and Omicron (BA.5) variants. **Results:** Vaccinated animals developed robust, TH1-biased IgG responses specific to the nucleocapsid protein. Following SARS-CoV-2 challenge, immunized hamsters exhibited reduced weight loss, lower oropharyngeal and lung viral titers, and improved lung pathology scores compared with unvaccinated controls. **Conclusion:** These findings support the potential of this universal vaccine to provide broad protection against current and future SARS-CoV-2 variants, with minimal need for updating.

## 1. Introduction

Coronavirus Disease 2019 (COVID-19), caused by severe acute respiratory syndrome coronavirus 2 (SARS-CoV-2) [1], a β-coronavirus closely related to human SARS-CoV [2], has resulted in substantial morbidity and mortality, with over 778 million cases and 7 million deaths globally [3]. Unlike the human β-coronavirus (β-hCoV) disease SARS, which occurred in 2002–2003 and was contained relatively quickly, and β-hCoV disease Middle East Respiratory Syndrome (MERS), which presented in 2012 and has remained geographically restricted [4], COVID-19 spread globally. In the process, SARS-CoV-2 original strain (Wuhan-Hu1) evolved rapidly with Variants of Concern such as Alpha (B.1.17), Beta (B.1.351), Gamma (B.1.1.28.1), Delta (B.1.617.2), and Omicron (BA.1, BA.2, BA.4/BA.5) emerging independently from one another in different parts of the world [5,6]. The Delta variant is highly infectious and has a greater capacity for infection and replication than the early strains [7,8]. The nucleocapsid protein mutations (R203M/D377Y) in the Delta variant promote viral replication and lung injury in the host [9]. In contrast, the Omicron variant has attenuated replication and pathogenicity and increased transmissibility due to its vaccine-escape mutations [10,11,12].

Like other coronaviruses, SARS-CoV-2 possesses a large ~30,000-nucleotide RNA genome, which encodes four structural proteins (spike, membrane (M), envelope, and nucleocapsid (N) proteins), non-structural proteins (NSPs), and accessory proteins [13]. The licensed COVID-19 vaccines have focused on the spike protein and generating neutralizing antibodies that inhibit host cell infection. However, the spike protein mutates rapidly and thereby can resist neutralizing antibodies, leading to immune escape by emerging variants [14,15]. Largely for this reason, immunity to spike protein-based vaccines quickly wanes after vaccination, repeatedly prompting redesigned booster vaccines comprising mutated spike proteins to restore immunity, although other strategies have been described [16,17,18]. In contrast to spike protein-based vaccines, vaccines centered on more conserved SARS-CoV-2 proteins hold promise for greater durability of protective immune responses.

The SARS-CoV-2 M protein, N protein, and NSP 12 (RNA-dependent RNA polymerase (RdRp)) are conserved within SARS-CoV-2 and among β-hCoV, and prominently recognized by T cells and, especially in the case of the N protein, B-cells [19,20,21]. The M protein is the most abundant structural protein in the viral envelope, drives coronavirus assembly, and stimulates T-cell responses [22,23]. The N protein, also abundantly expressed by the virus, participates in RNA packaging, facilitates virion assembly, enhances virus transcription, and is highly immunogenic and associated with control of SARS-CoV-2 replication in the upper airway [24,25,26,27]. The RdRp protein catalyzes the synthesis of viral RNA and plays a central role in the replication and transcription cycle of SARS-CoV-2 [28,29].

T cell immunity to M, N, and RdRp are conserved among β-hCoV. Convalescent donor SARS-CoV-2-specific CD4^+^ T cells predominantly respond to M and N proteins, and most SARS-CoV-2-specific CD8^+^ T cell epitopes reside in ORF1ab and N proteins, cross-reacting with SARS-CoV epitopes [20]. SARS-CoV-2-reactive T cells are detected in individuals unexposed to COVID-19 but seropositive to α-hCoV NL63 and β-hCoV OC43, indicating cross-reactive T cell recognition [23,30]. T cell memory responses to M, N, and S proteins persisted for 9–11 years in patients who recovered from SARS [31], and these T cells can contribute to SARS-CoV-2 clearance [32]. Healthcare workers remaining seronegative after exposure to SARS-CoV-2 show high levels of RdRp-specific T cells [33]. Pre-existing β-hCoV RdRp- and N-specific T cells protect against SARS-CoV-2 in the absence of neutralizing antibodies [20,26,33]. The N protein, which is translocated to the surface of infected host cells also induces antibody-dependent cellular cytotoxicity (ADCC) [21], and hence engages both humoral and cellular immunity. These characteristics of M, N, and RdRp make them ideal antigens for inclusion in a universal COVID-19 vaccine candidate aimed at inducing broad and long-lasting immunity [34,35,36]. An N-protein based vaccine has been reported to be effective against COVID-19 in a clinical study [37].

Using the LVS Δ*capB* single-vector platform [38], we developed LVS Δ*capB*-vectored SARS-CoV-2 vaccines, including rLVS Δ*capB*/MN expressing the fusion protein of SARS-CoV-2 M and N proteins derived from the early Washington Strain of SARS-CoV-2 (2019-nCoV/USA-WA1/2020) [34]. rLVS Δ*capB*/MN administered intradermally, intranasally (IN), subcutaneously, or orally (PO) provides potent protection against severe COVID-19-like disease caused by the homologous Washington strain in Syrian hamsters [34,35]. Here, we describe the development of rLVS Δ*capB*::RdRp/MN and studies comparing it with rLVS Δ*capB*/MN for efficacy against respiratory challenge with heterologous SARS-CoV-2 Delta and Omicron late variants.

## 2. Methods

### 2.1. Ethical Statement

Animal experiments were conducted according to the guidelines of the Declaration of Helsinki and the protocols approved by the CSU Institutional Animal Care and Use Committee (IACUC) (protocol number 5740, approved 6 May 2023). No data collected from animals were excluded from analysis. Investigators were not blinded to treatment group during the animal experiments or data analysis, except histopathologic examination and scoring was conducted in a blinded manner. On arrival, mice were randomly placed in cages and housed in ventilated, HEPA exhaust-filtered racks in order of treatment group.

### 2.2. Viruses and Bacteria

SARS-CoV-2 virus Delta variant (hCoV-19/USA/CA-VRLC086/2021, B.1.617.2-AY.1 variant, BEI Resources, NR-55694) and Omicron variant (hCoV-19/USA/COR-22-063113/2022, BA.5 variant, BEI Resources, NR-58620) were acquired through the NIH NIAID Biodefense and Emerging Infections Research Resources Repository (BEI Resources), and passaged and stocked as described by us previously [34,35]. The virus titer was determined by plaque assay as described previously [34]. *F. tularensis* Live Vaccine Strain with a deletion in *capB* (LVS Δ*capB*) was constructed as described by us previously [39]. Live attenuated recombinant LVS Δ*capB* expressing a highly efficient plasmid-encoded fusion protein of SARS-CoV-2 membrane (M) and nucleocapsid (N) proteins (rLVS Δ*capB*/SARS-CoV-2 MN, abbreviated as rLVS Δ*capB*/MN) was constructed and characterized as described by us previously [34]. Live attenuated recombinant LVS Δ*capB* expressing RdRp from the chromosome at the deleted *capB* locus and a highly efficient plasmid-encoded MN fusion protein (rLVS Δ*capB*::SARS-CoV-2 RdRp/MN, abbreviated as rLVS Δ*capB*::RdRp/MN) were constructed as described below. Stocks of LVS Δ*capB* vector, rLVS Δ*capB*/MN, and rLVS Δ*capB*::RdRp/MN vaccine candidates were prepared on chocolate agar as we described previously [39]. Briefly, the bacteria were re-suspended in sterile normal saline with an adjusted absorbance at 540 nm of 0.005; the bacterial suspension was spread on chocolate agar (0.1 mL/plate) and grown to a lawn at 37 °C in a CO_2_ incubator for 3 days; and the bacteria were scraped into 30 mL normal saline, collected by centrifugation at 6000× *g* for 20 min, washed twice with sterile normal saline, and resuspended in a 20% glycerol–normal saline solution. Aliquots (0.5 mL) were stored at −80 °C until use. Bacterial titers were determined immediately prior to freezing and periodically thereafter by spotting 0.05 mL of 10-fold serial dilutions onto chocolate agar plates, with or without kanamycin (7.5 µg/mL). Plates were incubated at 37 °C for 3–5 days, after which colony-forming units (CFU) were enumerated. Lysates of bacterial stocks grown on agar plates were examined by Western blotting for heterologous SARS-CoV-2 protein expression as described below.

### 2.3. Hamsters

Naïve golden Syrian hamsters (*Mesocricetus auratus*), 8–9 weeks old, were purchased from Charles River Laboratories (Wilmington, MA, USA) and randomly assigned to experimental groups. Animals were acclimated for at least 1 week prior to the start of an experiment. Animals were maintained in a specific-pathogen-free animal facility prior to challenge with the virulent SARS-CoV-2 variants. After challenge with SARS-CoV-2 variants, animals were relocated to and maintained in a BSL3 facility at CSU and used according to protocols approved by the CSU IACUC. All animals were observed at least once daily; unanticipated adverse events following vaccination and challenge were not observed.

### 2.4. Proteins, Antibodies, and Heat-Inactivated Bacteria

Recombinant SARS-CoV-2 nucleocapsid (N) protein N-terminal RNA-binding domain with an N-terminal histidine tag, expressed in *Escherichia coli* (NR-53246), and guinea pig polyclonal anti-SARS coronavirus antibody (NR-10361) were obtained from BEI Resources. Monoclonal anti-FLAG M2 horseradish peroxidase (HRP)-conjugated antibody was purchased from Millipore Sigma (St. Louis, MO, USA). Heat-inactivated LVS Δ*capB* (HI-LVS) stocks were prepared as previously described [39].

### 2.5. Generation of rLVS ΔcapB::RdRp/MN COVID-19 Vaccine Candidate

Previously, we generated rLVS Δ*capB*/MN expressing the fusion protein of an N-terminal 3FLAG-8His tag, SARS-CoV-2 M protein (QIH55224, 222 AA), a GGSG linker, and the N protein (QIH55228, 419 AA) from a highly efficient pFNL-derived plasmid DNA [34,35]; the M and N proteins were derived from SARS-CoV-2 WA1/2020 strain (GenBank: MT576556.1).

To construct rLVS Δ*capB* expressing SARS-CoV-2 RdRp, we purchased a synthesized DNA codon-optimized for expression in *Francisella tularensis* LVS from Atum (Newark, CA, USA). The DNA encodes a fusion protein comprising two RdRp T-cell epitopes (T123L131 (TL9) and S239L247 (SL9)) [20], the c-terminal 553 amino acids of RdRp (YP_009725307), and an 8His-3FLAG tag (combined 606 amino acids, 69 kDa, abbreviated as RdRp). We engineered the purchased DNA downstream of a strong *Francisella tularensis bacterioferritin* promoter (*bfr*) and a Shine-Dalgarno sequence, flanked by DNAs homologous to the upstream and downstream regions of the *capB* gene in the LVS Δ*capB* chromosome, into the multiple cloning sites of the modified pJC84 integration plasmid containing a kanamycin resistance gene and a sucrose suicide gene [40], by DNA assembly (NEBiulder). The DNA sequences of the molecular constructs were screened by restriction analysis and verified by whole plasmid nucleotide sequencing. The resultant recombinant integration plasmid DNA, pSG174, was integrated into the *capB* locus of *Francisella tularensis* LVS Δ*capB* via allelic exchange. Selection was performed on chocolate agar supplemented with kanamycin (7.5 µg/mL), followed by counter-selection on chocolate agar containing 8% sucrose to isolate marker-free recombinant strains (designated rLVS Δ*capB*::RdRp). Chromosomal integration of the RdRp antigen expression cassette at the *capB* locus was confirmed by polymerase chain reaction (PCR) and nucleotide sequencing. Subsequently, we introduced a highly efficient pFNL-derived shuttle plasmid DNA carrying an antigen cassette for the 3FLAG-8His-MN fusion protein into rLVS Δ*capB*::RdRp to generate rLVS Δ*capB*::RdRp/MN, expressing RdRp plus MN. These constructs were verified for SARS-CoV-2 protein expression by Western blotting using monoclonal anti-FLAG M2 HRP antibody and polyclonal anti-SARS-CoV antibody as described below.

### 2.6. Efficacy Study in Hamsters

Animals (8 per group, half male, half female) were immunized and challenged as described previously; group size was based on previous experiments that yielded statistically significant results [34,35]. Briefly, in the Delta challenge experiment, animals (total of 56) were immunized intranasally (IN) or orally (PO) three times, 3 weeks apart (Week 0, 3, and 6), with approximately 2 × 10^6^ CFU IN or 3 × 10^9^ CFU PO of rLVS Δ*capB*/MN or rLVS Δ*capB*::RdRp/MN vaccines diluted in 0.02 mL (IN) or 0.2 mL (PO) sterile phosphate-buffered saline (PBS), respectively. In the Omicron challenge experiment, animals (total of 56) were immunized IN or PO two times, 3 weeks apart (Week 0 and 3), with 2 × 10^6^ CFU IN or 3 × 10^9^ CFU PO with each of the two vaccines. Hamsters immunized PO were administered 1 × 10^9^ CFU 3 times (Monday-Wednesday-Friday) each week of vaccination. Thirty minutes prior to PO immunization, the animals were given 0.5 mL of 10% (*W*/*V*) sodium bicarbonate by gavage to neutralize gastric acid. Unvaccinated hamsters and hamsters vaccinated with equivalent IN or PO doses of LVS Δ*capB* (vector) served as controls. Vaccine back-titration was performed on chocolate agar on the same day of vaccination, confirming the above-described immunization doses. Blood was collected one week prior to the first immunization (Week-1), 2 weeks after each immunization, and one week prior to challenge to assess antibody responses; the sera were heat-inactivated at 56 °C for 30 min.

All animals were challenged IN at Week 10 (4 weeks after the third immunization in the Delta challenge experiment and 7 weeks after the second immunization in the Omicron challenge experiment) with approximately 1.1 × 10^4^ plaque forming units (pfu) of SARS-CoV-2 Delta variant (hCoV-19/USA/CA-VRLC086/2021, B.1.617.2-AY.1 variant, NR-55694) or 8.9 × 10^3^ pfu of SARS-CoV-2 Omicron variant (hCoV-19/USA/COR-22-063113/2022, BA.5 variant, NR-58620) under light anesthesia with ketamine-xylazine. Virus diluted in PBS was administered via pipette into the nares (100 µL total, ~50 µL/nare); animals were observed until fully recovered from anesthesia. Virus back-titration was performed on Vero E6 cells immediately following inoculation, confirming the above-described challenge doses. Animals were transferred to ABSL-3 containment facilities 6–8 days prior to viral challenge. After challenge, animals were monitored daily for clinical signs of disease, including fever, weight loss, and nasal discharge. Oropharyngeal swabs were collected on Days 1, 2, and 3 post-challenge for viral titration. In each experimental group, four animals were euthanized on Day 3 post-challenge (acute phase), when peak viral replication occurs, to assess viral titers in cranial and caudal lung lobes. The remaining animals were euthanized on Day 7 post-challenge (subacute phase) for histopathological evaluation, which is most pronounced at this time point. Despite the development of significant viral shedding, pulmonary viral loads, weight loss, and lung pathology, none of the hamsters became overtly ill following challenge, and humane endpoints were not utilized.

### 2.7. Histopathology Assessment

The histopathology assessment of lung tissues from hamsters obtained at 7 days post-challenge was performed as described by us previously by a single veterinary pathologist blinded to the identity of the vaccine groups [34,35]. Briefly, lung tissues from hamsters were preserved in 10% buffered formalin for a duration of 7 to 14 days, followed by paraffin embedding. Thin sections were then prepared and stained using hematoxylin and eosin. A single board-certified veterinary pathologist, blinded to the treatment groups, examined the slides. Histopathological scoring was conducted separately for cranial and caudal lung regions, assessing parameters including lesion extent, bronchitis, alveolitis, pneumocyte hyperplasia, vasculitis, and interstitial inflammation. Each parameter was rated on a scale of 0–4 or 0–5, and scores from each lung region were totaled.

### 2.8. Virus Assay

Virus titration was conducted using oropharyngeal swabs collected on Days 1, 2, and 3 post-challenge, as well as lung tissue samples (cranial and caudal regions) harvested on Day 3 post-challenge by double-overlay plaque assay on Vero E6 cells, following established protocols. In brief, fluid samples or homogenized tissues were serially diluted in Tris-buffered Minimum Essential Medium (MEM) supplemented with 1% bovine serum albumin (BSA), then applied to confluent Vero E6 cell monolayers in 6-well plates. After a 45 min incubation at 37 °C, each well was overlaid with 2 mL of MEM containing 2% fetal bovine serum and 0.5% agarose. Following 24–30 h of incubation at 37 °C in a 5% CO_2_ atmosphere, a second overlay—identical in composition but including neutral red dye—was added. Plaques were enumerated 48–72 h post-infection using a lightbox as described by us previously [34,35].

### 2.9. Enzyme-Linked Immunosorbent Assay (ELISA)

Hamster sera were assayed for IgG and subclass antibodies specific to SARS-CoV-2 N protein and HI-LVS antigens by ELISA as described previously [34,35,39]. Briefly, 96-well high-binding plates (Corning, Corning, NY, USA) were coated overnight at 4 °C with 0.1 mL of N protein solution (1 µg/mL) prepared in 50 mM carbonate/bicarbonate buffer (NaHCO_3_/Na_2_CO_3_). After removing unbound antigen, wells were blocked with Blocker Casein in PBS (Thermo Scientific, Waltham, MA, USA) for 1 h at room temperature. Serum samples were initially diluted 1:20 and then serially diluted three-fold in PBS containing 1% bovine serum albumin. These dilutions were incubated on the antigen-coated plates for 90 min, followed by a 90 min incubation with horseradish peroxidase (HRP)-conjugated goat anti-hamster IgG (ThermoFisher), or with mouse anti-hamster IgG1 (clone SB139A, Southern Biotech, Birmingham, AL, USA), or IgG2/3 (clone SB139e, Southern Biotech) antibodies at a 1:1000 dilution. Between each incubation step, plates were washed three times with PBS containing 0.05% Tween-20. Subsequently, 100 µL of tetramethylbenzidine (TMB) substrate in peroxide solution was added to each well and allowed to react for 15–20 min. The enzymatic reaction was terminated by adding 100 µL of 2 M sulfuric acid, and absorbance was measured at 450 nm using a multiscan microplate reader (TiterTek, Huntsville, AL, USA). Antibody titers were expressed as the log_10_ of the reciprocal of the highest serum dilution yielding an optical density (OD) exceeding the mean OD of sham-immunized controls plus three standard deviations. Results are reported as mean endpoint titers with standard error of the mean (SEM).

### 2.10. Statistics

Log scale mean pfu per swab and mean weight change was compared across vaccine group and time using a two-way (4 × 3) repeated measure (mixed) analysis of variance model. A repeated measure model is needed since observations on the same animal over day (time) are not independent. Normal quantile plots and the Shapiro–Wilks test confirm that the data (residual errors) have a normal distribution on this log scale. Under the model, comparisons of variances and absolute residual errors by group and day showed that the variance was not homogeneous across day or vaccine group. This was confirmed by a significant Brown–Forsythe test for comparing variances. Therefore, the model allowed the variance across days 1, 2, and 3 and/or across vaccine group to change. Mean log pfu per gram and mean pathological score was compared across the four vaccine groups using a one-way analysis of variance model with constant variance. Mean endpoint titers of pre- and post-vaccination serum IgG and its subclasses were compared among groups using two-way ANOVA with Tukey’s test (GraphPad Prism 10.4.0, San Diego, CA, USA). In Experiment 1, the correlation between mean anti-N protein serum IgG antibody endpoint titer at Week 5 and mean log10 oropharyngeal swab viral titer at Day 2 after Delta variant challenge was computed using linear regression. In Experiment 2, the correlation between mean anti-N protein serum IgG antibody endpoint titer at Week 5 and mean weight loss at Day 6, mean log10 oropharyngeal viral titers at Day 1, and mean lung histopathological score at Day 7 after Omicron challenge were computed using linear regression (Prism 10.4.0).

## 3. Results

### 3.1. Construction and Verification of rLVS ΔcapB:RdRp/MN Vaccine Candidate

**Figure 1:**
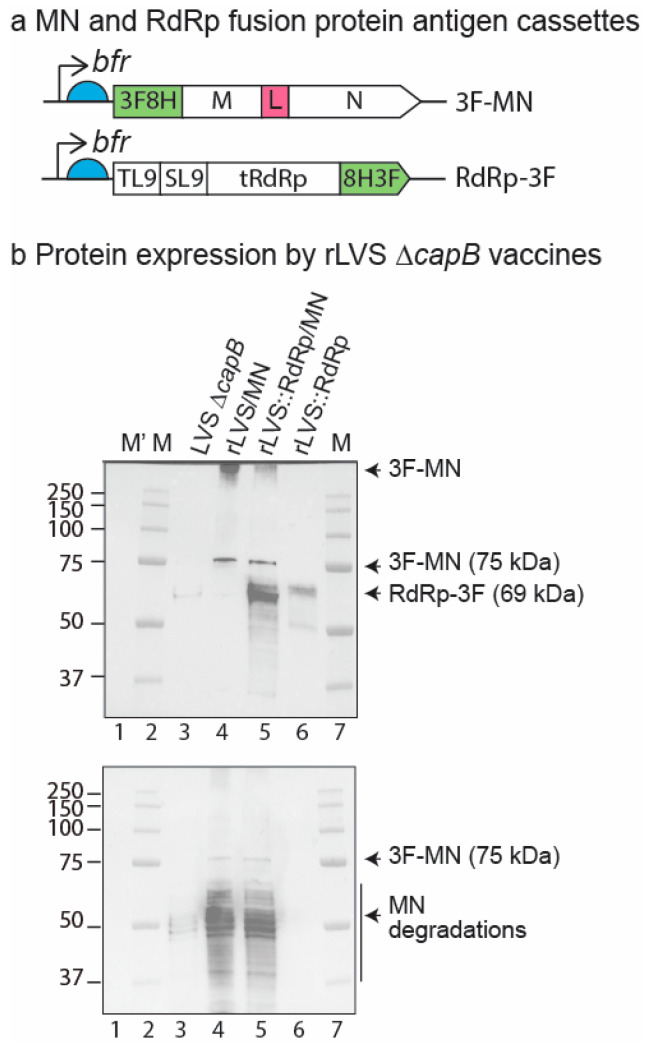
Protein expression of rLVS Δ*capB* vaccines expressing MN and RdRp. (**a**) Diagram of MN and RdRp antigen expression cassettes. The full-length M and N proteins of SARS-CoV-2 linked by a “GGSG” linker (L) with an N-terminal 3FLAG-8His (3F8H) (abbreviated as 3F-MN) is downstream of the *F. tularensis bacterioferritin* (*bfr*) promoter (thin black arrow) and a Shine–Dalgarno sequence (light blue half circle); similarly, a fusion protein of two conserved T cell epitopes (TL9 and SL9) of SARS-CoV-2 RdRp, the C-terminal 553 amino acids of RdRp (tRdRp), and a C-terminal 8His-3FLAG (8H3F) tag (abbreviated as RdRp-3F) is downstream of *bfr* promoter and Shine–Dalgarno sequence. (**b**) Protein expression by rLVS Δ*capB* vaccines. Total bacterial lysates of LVS Δ*capB* vector (lane 3), rLVS Δ*capB*/MN (lane 4), rLVS Δ*capB*::RdRp/MN (lane 5), and rLVS Δ*capB*::RdRp (lane 6) were analyzed by SDS-PAGE and Western blotting with monoclonal antibody to FLAG tag, which readily detected the full length 3F-MN (75 kD) and RdRp-3F (69 kDa) proteins, indicated by arrows to the right of the panel. Aggregates of 3F-MN protein were also detected at the top of lanes 4 and 5 and indicated by an arrow to the right of the panel (top panel). The blot was re-probed with anti-SARS-CoV guinea pig polyclonal antibody (BEI Resources, NR-10361), which readily detected the full-length MN (~ 75 kDa, less abundant) and the highly abundant breakdown products of MN protein, indicated by arrows and a vertical line to the right of the protein bands (bottom panel). The sizes of the molecular weight markers (M, M’) are labeled to the left of the panels. Unstained standards (M’, lane 1) are visible only on the stain-free gel (Supplementary Figure S1, panel a), while pre-stained standards (M) are invisible on the stain-free gel (Supplementary Figure S1, panel a) but visible in merged colorimetric and chemiluminescence images of the Western blots (top and bottom panels, lanes 2, 7).

Previously, we developed rLVS Δ*capB*/MN expressing the MN fusion protein from a highly efficient pFNL-derived plasmid DNA [34,35]. To broaden the antigenic pool, we constructed rLVS Δ*capB*::RdRp/MN, which expresses RdRp from an antigen expression cassette integrated at the deleted *capB* locus in the chromosome and the MN fusion protein from the shuttle plasmid as with rLVS Δ*capB*/MN. The RdRp expressed by the rLVS Δ*capB*::RdRp/MN comprises a fusion protein of two conserved T cell epitopes (T123L131 (TL9) and S239L247 (SL9)) of SARS-CoV-2 RdRp, the C-terminal 553 amino acids of RdRp [20,28,29], and an 8His-3FLAG tag (combined 606 amino acids, 69 kDa) downstream of a potent *Francisella tularensis bfr* promoter from the deleted *capB* locus in the chromosome (Figure 1, a) [34]. Epitope TL9-reactive T cells have strong cross-activity with β-hCoVs SARS-CoV and MERS-CoV and human α-hCoVs 229E and NL63, and are associated with reduced disease severity [20]. As with the recombinant SARS-CoV-2 proteins expressed by rLVS Δ*capB*/MN, the RdRp, M, and N proteins are derived from the early Washington strain of SARS-CoV-2. As shown in Figure 1b and Supplementary Figure S1, in contrast to the LVS Δ*capB* vector (Figure 1b, lane 3), rLVS Δ*capB*/MN expresses the 75-kDa full length MN fusion protein with an N-terminal 3FLAG-8His tag, detected by a monoclonal antibody to FLAG (Figure 1b, top panel, lane 4), and the full-length MN and its breakdown products, detected by a polyclonal antibody to SARS-CoV (Figure 1, panel b, bottom panel, lane 4). rLVS Δ*capB*::RdRp/MN expresses the 69-kDa RdRp with a C-terminal 8His-3FLAG tag as well as the 75-kDa MN (Figure 1b, top panel, lane 5) and its breakdown products (Figure 1b, bottom panel, lane 5). rLVS Δ*capB*::RdRp, the parental strain of rLVS Δ*capB*::RdRp/MN, expresses only RdRp, but not MN (Figure 1b, top and bottom panels, lane 6).

### 3.2. Study of Vaccine Efficacy Against SARS-CoV-2 Delta Variant Challenge in the Hamster Model—Experiment 1

**Figure 2:**
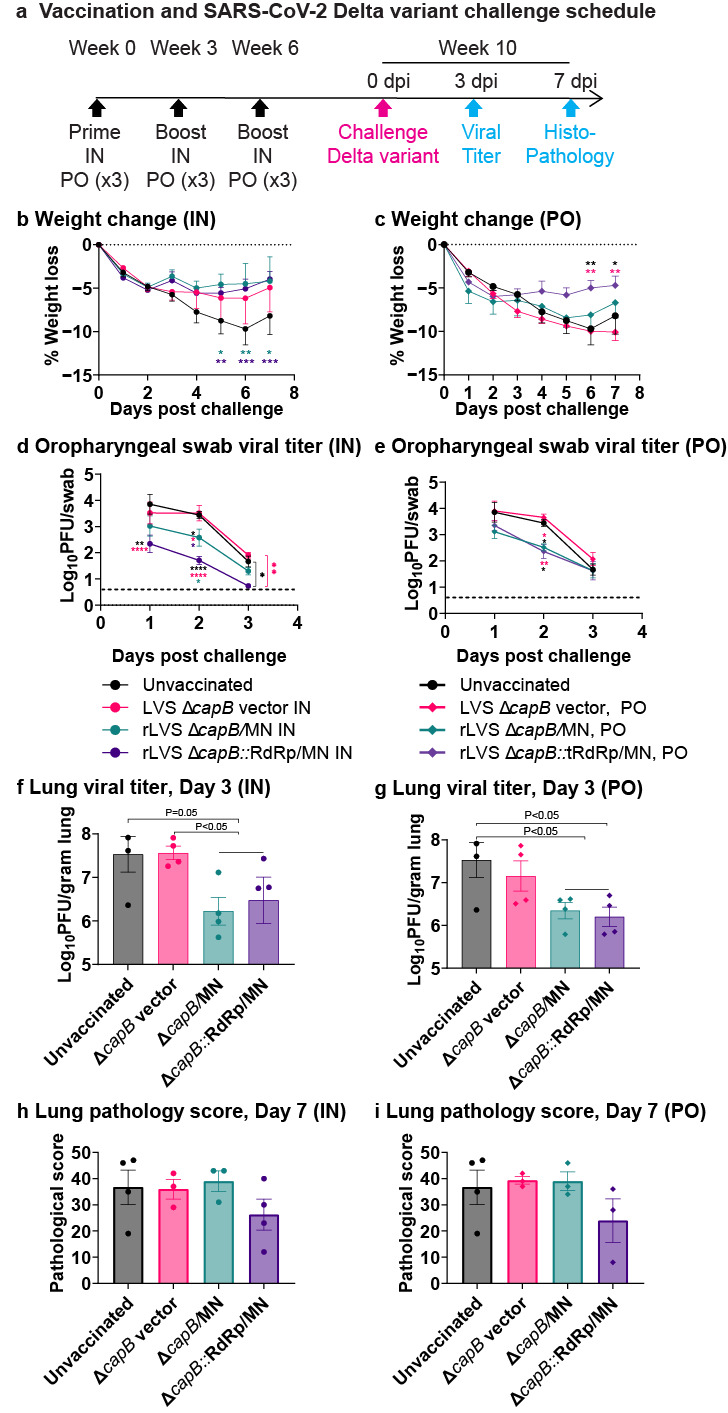
Efficacy of rLVS Δ*capB* COVID-19 vaccines against respiratory challenge with the SARS-CoV-2 Delta variant—Experiment 1. Golden Syrian hamsters (8/group, half male, half female; 7 groups for total of 56 animals) were immunized three times, 3 weeks apart at Weeks 0, 3, and 6, intranasally (IN) with 2 × 10^6^ CFU or orally (PO) with 3 × 10^9^ CFU rLVS Δ*capB*/MN or rLVS Δ*capB*::RdRp/MN vaccine candidates. Unvaccinated hamsters and hamsters immunized with equivalent IN or PO doses of the LVS Δ*capB* vector served as controls. Four weeks after the last immunization (Week 10), we challenged all of the hamsters IN with 1.1 × 10^4^ pfu of SARS-CoV-2 Delta variant (B.1.617.2-AY.1) and observed them daily after for clinical indicators of disease progression, including changes in body weight (eight animals/per group from Day 1 to Day 3 and four animals/group from Day 4 to Day 7). Viral titers were assessed in oropharyngeal swabs (eight animals/group) collected on Days 1, 2, and 3 post-challenge and in nasal turbinates and lung tissues (four animals/group) collected on Day 3 post-challenge. Histopathological changes were examined in lung tissues (cranial and caudal portions) (four animals/group) at Day 7 post-challenge. (**a**) Immunization and challenge schedule. (**b**,**c**) Weight loss post-challenge in hamsters immunized IN (**b**) or PO (**c**). Data are mean % weight loss from 0 days post-challenge. (**d**,**e**) Viral titers in oropharyngeal swabs Days 1–3 post-challenge in hamsters immunized IN (**d**) or PO (**e**). (**f**,**g**) Viral titer in the cranial and caudal lung homogenates on Day 3 post-challenge in hamsters immunized IN (**f**) or PO (**g**). The horizontal line above the two vaccine groups (rLVS Δ*capB*/MN and rLVS Δ*capB*::RdRp/MN) represents these two groups combined into one group of eight animals for statistical analysis. (**h**,**i**) Lung histopathology scores at Day 7 post-challenge in hamsters immunized IN (**h**) or PO (**i**). Data are means ± SEM. Mean weight loss and log scale mean pfu per swab post-challenge were compared using a two-way repeated measure (mixed) analysis of variance model. Log scale mean pfu per gram turbinate and lung tissue and mean histopathological scores in cranial and caudal lungs were compared using one-way analysis of variance model. * *p* < 0.05; ** *p* ≤ 0.01, ***, *p* < 0.001, ****, *p* < 0.0001 comparing the group to which the asterisks are adjacent with the group bearing the same color as the asterisks. There were no significant differences in lung pathology scores among different groups (**h**,**i**).

To investigate the cross-protective efficacy of rLVS Δ*capB*::RdRp/MN and rLVS Δ*capB*/MN against respiratory challenge with the heterologous SARS-CoV-2 Delta variant, we immunized hamsters in groups of eight (half male, half female), three times, 3 weeks apart, IN with 2 × 10^6^ colony forming units (CFU) or PO Monday-Wednesday-Friday of each vaccination week with 1 × 10^9^ CFU of rLVS Δ*capB*/MN or rLVS Δ*capB*::RdRp/MN and challenged them 4 weeks later with the SARS-CoV-2 Delta variant (B.1.617.2-AY.1) (Figure 2a). Unvaccinated hamsters and hamsters vaccinated with the LVS Δ*capB* vector served as controls.

As shown in Figure 2, hamsters immunized IN with rLVS Δ*capB*/MN or rLVS Δ*capB*::RdRp/MN exhibited significantly less weight loss compared with unvaccinated controls and/or vector-immunized controls (Figure 2b, Supplementary Table S1). Specifically, the unvaccinated hamsters lost a maximum of approximately 10% of their weight, similar to unvaccinated hamsters challenged with one of the earliest SARS-CoV-2 isolates, the WA1/2020 isolate [34]. Hamsters vaccinated IN with rLVS Δ*capB*/MN and rLVS Δ*capB*::RdRp/MN lost a maximum of 5% of their weight on Day 4 post-challenge and limited weight loss to 4% at Day 7 post-challenge, significantly less weight loss than the unvaccinated animals (*p* < 0.05–*p* < 0.001) (Figure 2b, Supplementary Table S1). Of note, hamsters immunized with the vector control also had reduced weight loss compared with unvaccinated animals after Delta variant challenge; however, the difference was not statistically significant. Hamsters vaccinated PO with rLVS Δ*capB*::RdRp/MN had approximately 6% maximum weight loss at Day 3 post-challenge and started gaining weight thereafter; their weight loss was significantly less than that of the unvaccinated hamsters and hamsters vaccinated PO with the vector (*p* < 0.05–*p* ≤ 0.01); hamsters vaccinated PO with rLVS Δ*capB*/MN also had reduced weight loss compared with unvaccinated animals, although the difference was not statistically significant (Figure 2c, Supplementary Table S2). Notably, the addition of RdRp to the MN vaccine further reduced weight loss in PO-immunized animals.

Hamsters vaccinated IN or PO with rLVS Δ*capB*/MN or rLVS Δ*capB*::RdRp/MN also had significantly lower viral titers in oropharyngeal swabs (*p* < 0.05–*p* < 0.0001) as well as lower viral titers in the lungs, a difference that was significant for animals immunized PO with rLVS Δ*capB*::RdRp/MN (*p* < 0.05) (Figure 2d–g). We further compared the unvaccinated and vector control groups with LVS Δ*capB*/MN and rLVS Δ*capB*::RdRp/MN combined as a single vaccine group. Hamsters vaccinated IN and PO with the vaccines had significantly lower viral titers in the lung than unvaccinated and vector-vaccinated hamsters (*p* ≤ 0.05) (Figure 2f,g). Hamsters vaccinated IN or PO with rLVS Δ*capB*::RdRp/MN also had lower histopathological scores in the lungs than unvaccinated or vector-immunized animals (Figure 2h,i; Supplementary Table S3) at Day 7 post-challenge, although the differences were not statistically significant, possibly due to missing the peak of lung pathology, as weight loss was maximal at Day 6 post-challenge (Figure 2b,c). Of note, the addition of RdRp to the MN vaccine further reduced viral titers in oropharyngeal swabs after IN administration (*p* < 0.05) and reduced histopathological scores in lungs after both IN and PO administration, although the differences in histopathological scores did not reach statistical significance (Figure 2d,e,h,i). Hamsters vaccinated IN or PO with rLVS Δ*capB*/MN or rLVS Δ*capB*::RdRp/MN also had lower viral titers in the turbinates, but these differences were not statistically significant (Supplemental Figure S2a).

**Figure 3:**
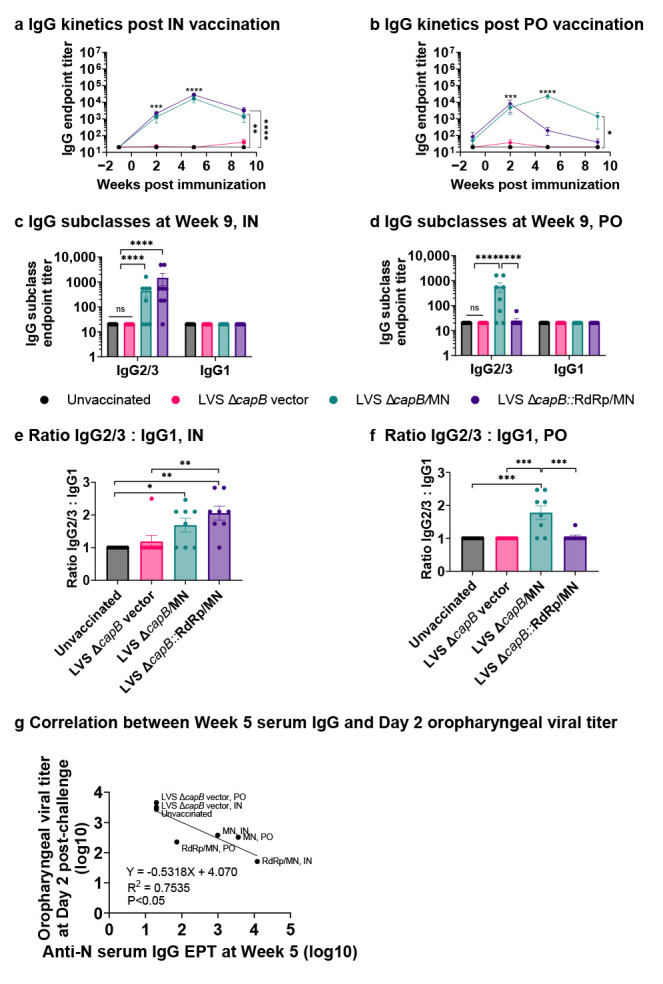
Humoral immune response—Experiment 1. Golden Syrian hamsters were immunized three times, 3 weeks apart at Week 0 and 3, IN with 2 × 10^6^ CFU or PO with 1 × 10^9^ CFU rLVS Δ*capB*/MN or rLVS Δ*capB*::RdRp/MN vaccine candidates. Unvaccinated hamsters and hamsters immunized with equivalent IN or PO doses of the LVS Δ*capB* vector served as controls. Hamsters were bled at Week 1 (one week prior to immunization), 2, 5, and 9 and sera assayed for IgG antibody specific to the SARS-CoV-2 N protein. (**a**,**b**) Kinetics of serum IgG specific to N protein. Sera were evaluated for IgG specific to N protein at Week 1, 2, 5, and 9 after IN (**a**) or PO (**b**) vaccination. (**c**,**d**) Serum IgG subclasses (IgG1 and IgG2/3) specific to N protein. Sera collected at Week 9 were evaluated for IgG subclasses specific to N protein after IN (**c**) or PO (**d**) vaccination. (**a**–**d**), black, unvaccinated; megenta, LVS Δ*capB* vector; green, LVS Δ*capB*/MN; and purple, LVS Δ*capB*::RdRp/MN. (**e**,**f**) Ratio of log_10_ endpoint titer of IgG2/3 to IgG1 after IN (**e**) and PO (**f**) vaccination at Week 9. (**a**–**f**) Data are mean ± SEM. *, *p* < 0.05, **, *p* < 0.01, ***, *p* < 0.001, and ****, *p* < 0.0001 by two-way or one-way ANOVA with Tukey’s multiple comparisons test (GraphPad Prism 10.4.0). (**g**) Correlation between mean log10 anti-N IgG endpoint titer (EPT) at Week 5 (**a**,**b**) and mean log10 oropharyngeal viral titer at Day 2 post-challenge (Figure 2d,e).

As expected, both IN and PO immunization with rLVS Δ*capB*/MN and rLVS Δ*capB*::RdRp/MN induced significantly elevated N-protein specific serum IgG compared with unvaccinated and vector-immunized animals (*p* < 0.01–0.0001) at two weeks after a single immunization at Week 0 (*p* < 0.0001), which peaked at Week 5 for both vaccines after an IN booster immunization and for the rLVS Δ*capB*/MN vaccine after a PO booster immunization (*p* < 0.0001) (Figure 3a,b). The IgG response for both vaccines was dominated by the TH1-type biased subclass IgG2/3 (Figure 3c–f). The anti-N IgG antibody endpoint titer at Week 5 (peak time) correlates with oropharyngeal viral titer at Day 2 post-challenge (*p* < 0.05) (Figure 3g).

Taken together, these results indicate that the addition of RdRp to the MN antigens expressed by the LVS Δ*capB* vector improves the immune protection of the vaccine against challenge with the SARS-CoV-2 Delta variant.

### 3.3. Study of Vaccine Efficacy Against SARS-CoV-2 Omicron Variant Challenge in the Hamster Model—Experiment 2

**Figure 4:**
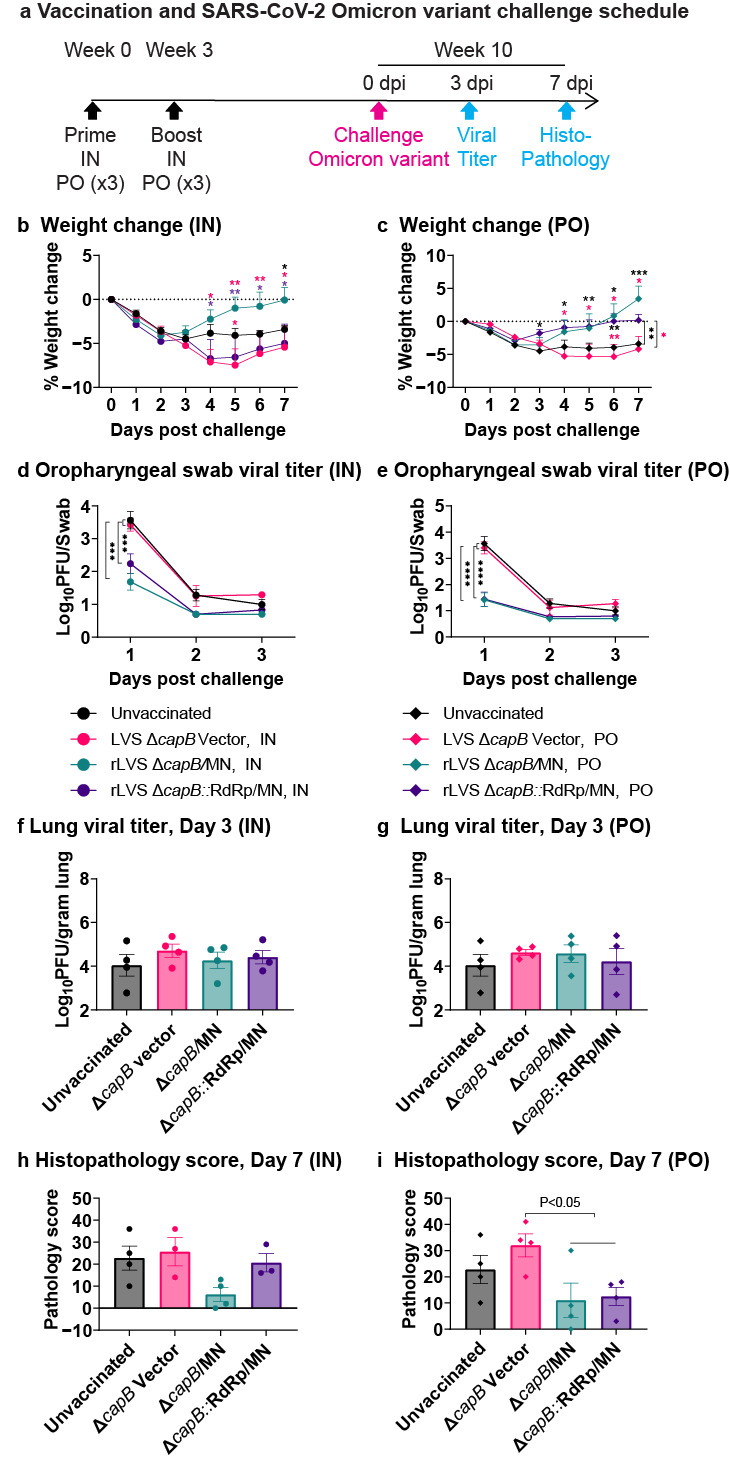
Efficacy of rLVS Δ*capB* COVID-19 vaccines against respiratory challenge with the SARS-CoV-2 Omicron variant—Experiment 2. Golden Syrian hamsters (8/group, half male, half female; 7 groups for total of 56 animals) were immunized twice, at Week 0 and 3, IN with 2 × 10^6^ CFU or PO with 3 × 10^9^ CFU of rLVS Δ*capB*/MN or rLVS Δ*capB*::RdRp/MN vaccine candidates. Unvaccinated hamsters and hamsters immunized with equivalent IN or PO doses of the LVS Δ*capB* vector served as controls. Seven weeks after the last immunization (Week 10), all of the hamsters were challenged IN with 8.9 × 10^3^ pfu Omicron Variant (BA.5). Body weight and clinical signs were monitored daily following viral challenge. Oropharyngeal swabs were collected on Days 1, 2, and 3 post-infection to assess viral titers using plaque assays. Four animals per group were euthanized on Day 3 post-challenge for analysis of viral loads in nasal turbinates and lung tissues. The remaining animals (*n* = 4/group) were euthanized on Day 7 post-challenge for histopathological assessment of lung tissue. (**a**) Immunization and challenge schedule. (**b**,**c**) Weight loss post-challenge in hamsters immunized IN (**b**) or PO (**c**). Data are mean % weight loss from 0 days post-challenge. (**d**,**e**) Viral titer in the oropharyngeal swabs Days 1–3 post-challenge in hamsters immunized IN (**d**) or PO (**e**). (**f**,**g**) Viral titer in the cranial and caudal lung homogenates Day 3 post-challenge in hamsters immunized IN (**f)** or PO (**g**). (**h**,**i**) Lung histopathology scores at Day 7 post-challenge in hamsters immunized IN (**h**) or PO (**i**). The horizontal line above the two vaccine groups (rLVS Δ*capB*/MN and rLVS Δ*capB*::RdRp/MN) in panel (**i**) represents these two groups combined into one group of eight animals for statistical analysis. Data are means ± SEM. Mean weight loss and log scale mean pfu per swab post-challenge were compared using a two-way repeated measure (mixed) analysis of variance model. Log scale mean pfu per gram turbinate or lung tissue and mean histopathological scores in cranial and caudal lungs were compared using one-way analysis of variance model. * *p* < 0.05; ** *p* < 0.01, ***, *p* < 0.001, ****, *p* < 0.0001, comparing the group to which the asterisks are adjacent with the group bearing the same color as the asterisks. Differences in lung viral titers between groups vaccinated IN (**f**) or PO (**g**) or in pathology scores between groups vaccinated IN (**h**) were not statistically significant.

Subsequently, we investigated whether rLVS Δ*capB*::RdRp/MN and rLVS Δ*capB*/MN cross-protect animals against respiratory challenge with the heterologous SARS-CoV-2 Omicron variant. To do so, we immunized hamsters as described above, except only two times rather than three (N-specific IgG antibody usually peaked at Week 5 after two immunizations (Figure 3a,b)), at Weeks 0 and 3, IN with 2 × 10^6^ CFU or PO with 3 × 10^9^ CFU of rLVS Δ*capB*/MN or rLVS Δ*capB*::RdRp/MN and challenged them seven weeks later at Week 10 with 8.9 × 10^3^ pfu Omicron variant (BA.5) (Figure 4a). Unvaccinated hamsters and hamsters vaccinated with the LVS Δ*capB* vector at the same IN and PO doses served as controls. As shown in Figure 4, hamsters immunized IN with rLVS Δ*capB*/MN had significantly reduced weight loss compared with hamsters immunized with the vector control (*p* < 0.05–0.01), and hamsters immunized PO with rLVS Δ*capB*/MN or rLVS Δ*capB*::RdRp/MN had significantly reduced weight loss compared with hamsters immunized with the vector control or unvaccinated animals (*p* < 0.05–0.001) (Figure 4b,c, Supplementary Tables S4 and S5). Hamsters immunized IN or PO with rLVS Δ*capB*/MN or rLVS Δ*capB*::RdRp/MN also had significantly lower viral titers than unimmunized or vector-immunized animals in oropharyngeal swabs (*p* < 0.001–0.0001) (Figure 4d,e) as well as lower viral titers in turbinates (Supplementary Figure S2b), but not in the lung (Figure 4f,g), and lower histopathological scores in the lung (Figure 4h,i) (Supplementary Table S6). When we further compared the unvaccinated and vector control groups with LVS Δ*capB*/MN and rLVS Δ*capB*::RdRp/MN combined as a single vaccine group, we found that hamsters vaccinated PO with the vaccines had significantly lower histopathological scores in the lung than hamsters vaccinated with the vector (*p* < 0.05) (Figure 4i). Viral titers of vaccinated and control animals in the lungs were similar (Figure 4f,g). Of note, in unvaccinated and vector-immunized animals, viral titers in the lungs and turbinates were much lower by ~3 logs after Omicron than Delta challenge and histopathological scores in these groups lower by ~one-third, reflecting the lower virulence of the Omicron vs. Delta variant.

**Figure 5:**
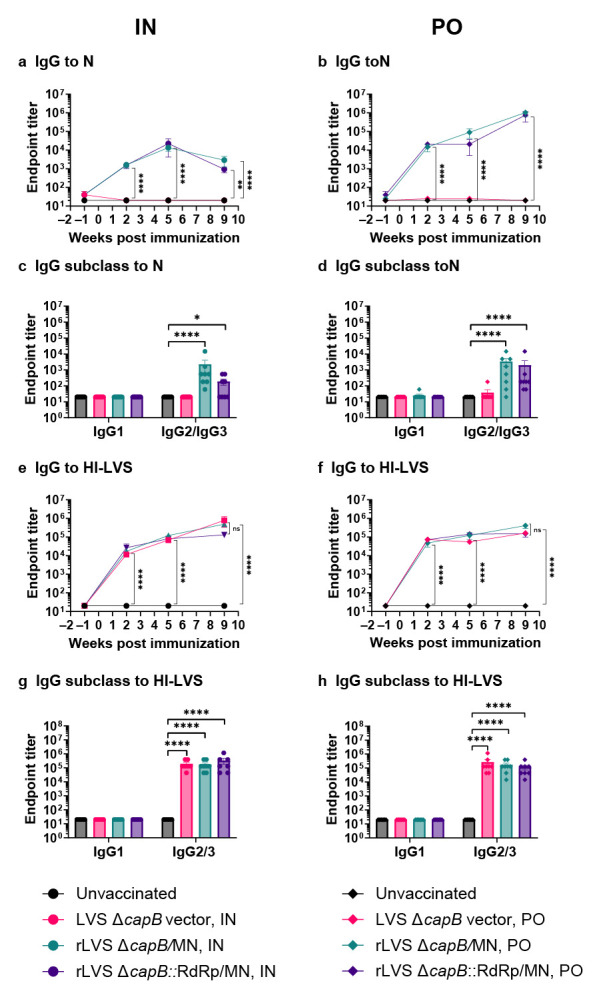
Humoral immune response—Experiment 2. Golden Syrian hamsters were immunized twice, 3 weeks apart at Week 0 and 3, IN with 2 × 10^6^ CFU or PO with 1 × 10^9^ CFU rLVS Δ*capB*/MN or rLVS Δ*capB*::RdRp/MN vaccine candidates. Unvaccinated hamsters and hamsters immunized with equivalent IN or PO doses the LVS Δ*capB* vector served as controls. Hamsters were bled at Week 1 (one week prior to immunization), 2, 5, and 9 and sera assayed for IgG antibody specific to the SARS-CoV-2 N protein and HI-LVS. (**a**,**b**) Kinetics of serum IgG specific to N protein. Sera were evaluated for IgG specific to N protein at Week -1, 2, 5, and 9 after IN (**a**) or PO (**b**) vaccination. (**c**,**d**) Serum IgG subclasses (IgG1 and IgG2/3) specific to N protein. Sera collected at Week 9 were evaluated for IgG subclasses specific to N protein after IN (**c**) or PO (**d**) vaccination. (**e**,**f**) Kinetics of serum IgG specific to lysates of HI-LVS. Sera were evaluated for IgG specific to HI-LVS at Week -1, 2, 5, and 9 after IN (**e**) or PO (**f**) vaccination. (**g**,**h**) Serum IgG subclasses (IgG1 and IgG2/3) specific to lysates of HI-LVS. Sera collected at Week 9 were evaluated for IgG subclasses specific to HI-LVS after IN (**g**) or PO (**h**) vaccination. (**a**–**h**) Data are mean ± SEM. *, *p* < 0.05, **, *p* < 0.01, and ****, *p* < 0.0001 by two-way ANOVA with Tukey’s multiple comparisons test (GraphPad Prism 10.4.0).

**Figure 6:**
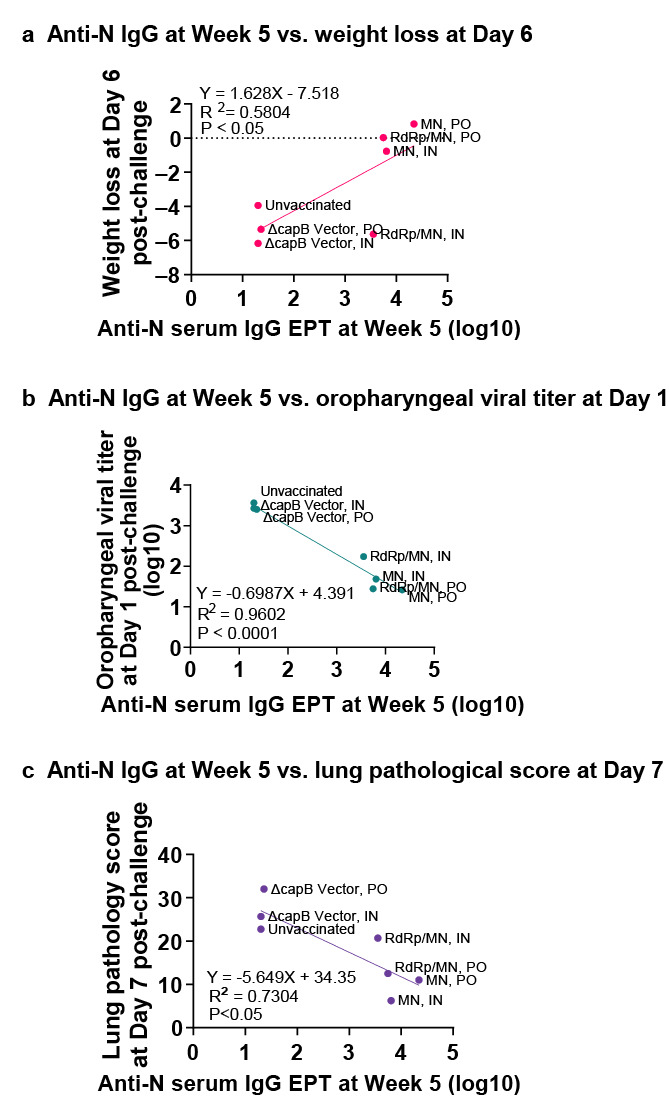
Correlate of protection—Experiment 2. Hamsters were immunized IN or PO as described in Figure 4a. Sera were evaluated for N protein specific IgG antibody as described in Figure 5. (**a**) Correlation between mean log10 anti-N IgG endpoint titer (EPT) at Week 5 (as shown in Figure 5a,b) and weight loss at Day 6 post-Omicron challenge (as shown in Figure 4b,c) for all groups (IN and PO). (**b**) Correlation between mean anti-N IgG EPT at Week 5 and mean log10 oropharyngeal viral titer at Day 1 post-Omicron challenge (as shown in Figure 4d,e) for all groups (IN and PO). (**c**) Correlation between mean anti-N IgG EPT at Week 5 and mean lung histopathology score on Day 7 post-challenge (sum of cranial and caudal lung as shown in Figure 4h,i) for all groups (IN and PO).

Hamsters immunized IN or PO with rLVS Δ*capB*/MN or rLVS Δ*capB*::RdRp/MN had elevated levels of serum IgG specific to the N protein (*p* < 0.0001 vs. unvaccinated or vector-immunized animals); antibody subclasses displayed a TH1-type bias reflected by dominance of subclass IgG2/3 (Figure 5a–d). Of note, PO immunization with the MN and RdRp/MN vaccines induced serum IgG to the N protein at greater levels than those induced by IN immunization, indicating robust immunity induced by the PO route (Figure 5a,b). Anti-N protein serum IgG endpoint titer at Week 5 correlates with weight loss at Day 6 post-challenge (Figure 6a), oropharyngeal viral titer at Day 1 post-challenge (Figure 6b), and lung histopathological score at Day 7 post-challenge (Figure 6c). The rLVS Δ*capB*/MN and rLVS Δ*capB*::RdRp/MN vaccines and the LVS Δ*capB* vector also induced serum IgG antibodies specific to heat-inactivated LVS (HI-LVS) with a TH1-type bias dominated by IgG2/3 (Figure 5e–h).

## 4. Discussion

In a study extending previous work with our single-vector platform rLVS Δ*capB* COVID-19 vaccines, we show that rLVS Δ*capB*/MN and rLVS Δ*capB*::RdRp/MN vaccines expressing relatively highly conserved proteins derived from the early Washington strain of SARS-COV-2, when administered either IN or PO, demonstrate robust cross-protection against heterologous Delta and Omicron SARS-CoV-2 variants, reducing disease severity and viral replication, despite several mutations in the M, N, and RdRp proteins in the Delta and Omicron variants (Supplementary Table S7). Adding RdRp improved outcomes against the Delta variant, possibly due to broader T-cell activation; however, differences between MN and RdRp/MN vaccines were not always statistically significant. The broader antigen repertoire provided by the conserved M, N, and RdRp proteins may provide more reliable cross-protection considering the challenge posed by rapidly mutating viral strains, and better enable the vaccines to remain effective against future variants.

The mRNA vaccines targeting the SARS-CoV-2 surface spike protein offer high initial efficacy (~90%), but efficacy wanes against variants, especially Omicron (efficacy drops to ~30–50% for infection prevention) [17], due to immune evasion. Our strategy of targeting relatively conserved M, N, and RdRp proteins avoids spike mutation-driven immune evasion, which substantially exceeds that of the M, N, and RdRp proteins (Supplementary Table S7), while eliciting N protein-dependent ADCC and T-cell responses that recognize and combat virus infected cells rather than preventing virus entry. The M, N, and RdRp’s high conservation (66–96% homology across β-hCoVs) positions these proteins as “universal” vaccine antigens, potentially effective against future pandemic variants. While our vaccines’ reliance on combatting infected host cells via ADCC and T-cell immunity rather than via blocking viral entry by virus neutralization might not prevent infection as effectively as spike-based vaccines, our vaccines may more reliably reduce disease severity. This aligns with studies showing the presence of M-, N-, and RdRp-specific T-cells correlate with milder disease, even in unvaccinated individuals exposed to β-hCoV [32,41,42].

In addition to targeting SARS-CoV-2, the LVS Δ*capB* single-vector platform has been utilized to generate potent broadly immunogenic vaccines against multiple other pathogens including the Select Agents of tularemia, anthrax, plague, and melioidosis [34,35,38,43,44,45]. Along with efficacy, the LVS Δ*capB* platform offers significant advantages in terms of safety, ease of administration, manufacture, and cost. Since the LVS Δ*capB* platform comprises a highly attenuated *capB* mutant of a vaccine (LVS) already administered to ~60 million people (LVS Δ*capB* is >10,000-fold less virulent than LVS when administered IN to highly susceptible mice [39]), it should be exceedingly safe. The rLVS Δ*capB* platform vaccines can be administered IN, PO, subcutaneously, or intradermally [34,35], making them easy to distribute, especially in resource-poor settings. Oral administration, in particular, which does not require the trained personnel needed to deliver parenteral vaccines, could dramatically improve immunization rates in low- and middle-income countries and may be attractive to many people with vaccine hesitancy stemming from fear of needles. LVS Δ*capB*-vectored vaccines are easily and inexpensively manufactured in simple broth culture without the need for extensive purification, and lyophilized, the vaccines can be stored and transported at refrigerator temperature; if prepared by vitrification, they potentially can be stored and transported at room temperature. These are significant advantages over other vaccine platforms that typically require complex and costly production and purification processes and, in the case of mRNA vaccines, storage at ultra-low temperatures.

## 5. Conclusions

Our studies show that LVS Δ*capB* single-vector platform COVID-19 vaccines, rLVS Δ*capB*/MN and rLVS Δ*capB*::RdRp/MN, expressing the fusion protein of the conserved M and N proteins alone or additionally with the RdRp protein, respectively, from an early strain of SARS-CoV-2, cross-protect golden Syrian hamsters against intranasal challenge with the Delta (B.1.617.2-AY.1) and Omicron (BA.5) variants. rLVS Δ*capB*::RdRp/MN, with its focus on multiple conserved antigen targets, makes it a promising candidate vaccine for long-term protection against evolving coronaviruses. Moreover, new potentially even longer-term universal rLVS Δ*capB*::RdRp/MN vaccines can easily be constructed using sequences of M, N, and RdRp proteins from currently circulating SARS-CoV-2 strains.

## Data Availability

The authors confirm that the data supporting the findings of this study are available within the article and its Supplementary Materials.

## Supplementary Materials

The following supporting information can be downloaded at: https://www.mdpi.com/article/10.3390/vaccines13060633/s1, Figure S1: Protein expression of rLVS ΔcapB vaccines expressing MN and RdRp; Figure S2: Viral titers in the turbinates of hamsters immunized and challenged with SARS-CoV-2 Delta and Omicron strain; Figure S3. Densitometry readings of each band in Supplementary Figure S1b using Image Lab software (Version 6.1.0. Build 7, Bio-Rad Laboratory); Figure S4. Densitometry readings of each band in Supplementary Figure S1c using Image Lab software; Table S1: Weight change post Delta variant challenge in IN-immunized hamsters; Table S2: Weight change post Delta variant challenge in PO-immunized hamsters; Table S3: Lung histopathological scores post Delta variant challenge; Table S4. Weight change post Omicron variant challenge in IN-immunized hamsters; Table S5: Weight change post Omicron variant challenge in PO-immunized hamsters; Table S6: Lung histopathological scores post Omicron variant challenge; Table S7: Amino acid differences between the M, N, and RdRp of the early SARS-CoV-2 isolates and the late Delta and Omicron variants.
