# Supplementary material for: Universal Bacterium-Vectored COVID-19 Vaccine Expressing Early SARS-CoV-2 Conserved Proteins Cross-Protects Against Late Variants in Hamsters
Source: Vaccines (Basel). 2025 Jun 12;13(6):633. doi: 10.3390/vaccines13060633 (PMC12197694; doi:10.3390/vaccines13060633)
Supplement: Supplementary file 1 [file vaccines-13-00633-s001.zip › vaccines-3676471-supplementary.pdf]

Supplementary Information

Universal bacterium-vectored COVID-19 vaccine expressing early SARS-CoV-2 conserved proteins cross-protects against late variants in hamsters

Qingmei Jia[1], Helle Bielefeldt-Ohmann[2], Saša Masleša-Galić[1], Richard A. Bowen[3] and Marcus A. Horwitz[1,*]

[1] Division of Infectious Diseases, Department of Medicine, 32-150 Center for Health Sciences, School of Medicine, University of California – Los Angeles, 10833 Le Conte Avenue, Los Angeles, CA 90095-1688

[2] Australian Infectious Diseases Research Centre, University of Queensland, St Lucia, Qld 4072, Australia

[3] Department of Biomedical Sciences, Colorado State University, Fort Collins, CO 80523

[*]Corresponding author. Email: MHorwitz@mednet.ucla.edu

Supplementary Figures S1-S4

Supplementary Tables S1-S7

Supplementary Figure S1

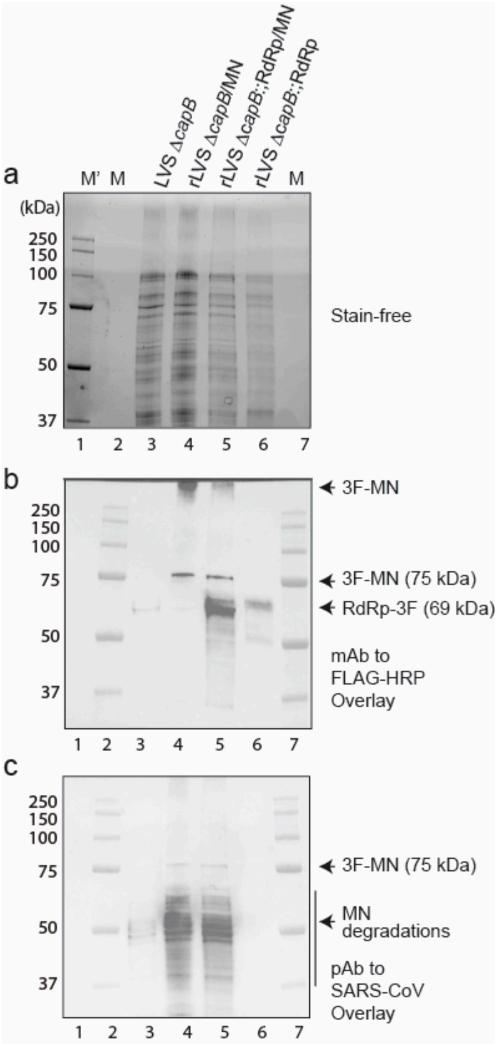

Supplementary Figure S1. Protein expression of rLVS Δ*capB* vaccines expressing MN and RdRp. Total bacterial lysates of LVS Δ*capB* vector (lane 3), rLVS Δ*capB*/MN (lane 4), rLVS Δ*capB*::RdRp/MN (lane 5) and rLVS Δ*capB*::RdRp (lane 6) were analyzed by SDS-PAGE and Western blotting. **a.** Stain free image of the protein gel. **b**. Western blotting analysis with monoclonal antibody to FLAG tag, which readily detected the full length 3FLAG-MN (75 kD) and RdRp-3FLAG (69 kDa) proteins, indicated by arrows to the right of the panel. Aggregates of

MN protein were also detected at the top of lanes 4 and 5 and indicated by an arrow to the right of the panel. **c.** Western blot analysis with anti-SARS-CoV guinea pig polyclonal antibody (BEI Resources, NR-10361), which readily detected the full-length MN (~ 75 kDa, less abundant) and the highly abundant breakdown products of MN protein, indicated by arrows and a vertical line to the right of the protein bands. The sizes of the molecular weight markers (M, M') are labeled to the left of the panels. Unstained standards (M', lane 1) are visible only on the stain-free gel (panel a), while pre-stained standards (M) are invisible on the stain-free gel (panel a) but visible in merged colorimetric and chemiluminescence images of the Western blots (panels b and c, lanes 2, 7). Panels b and c, replicates of Figure 1, b of main paper. Densitometry readings of major proteins bands in panels b and c are shown in Supplementary Figure S3 and S4, respectively.

Supplementary Figure S2

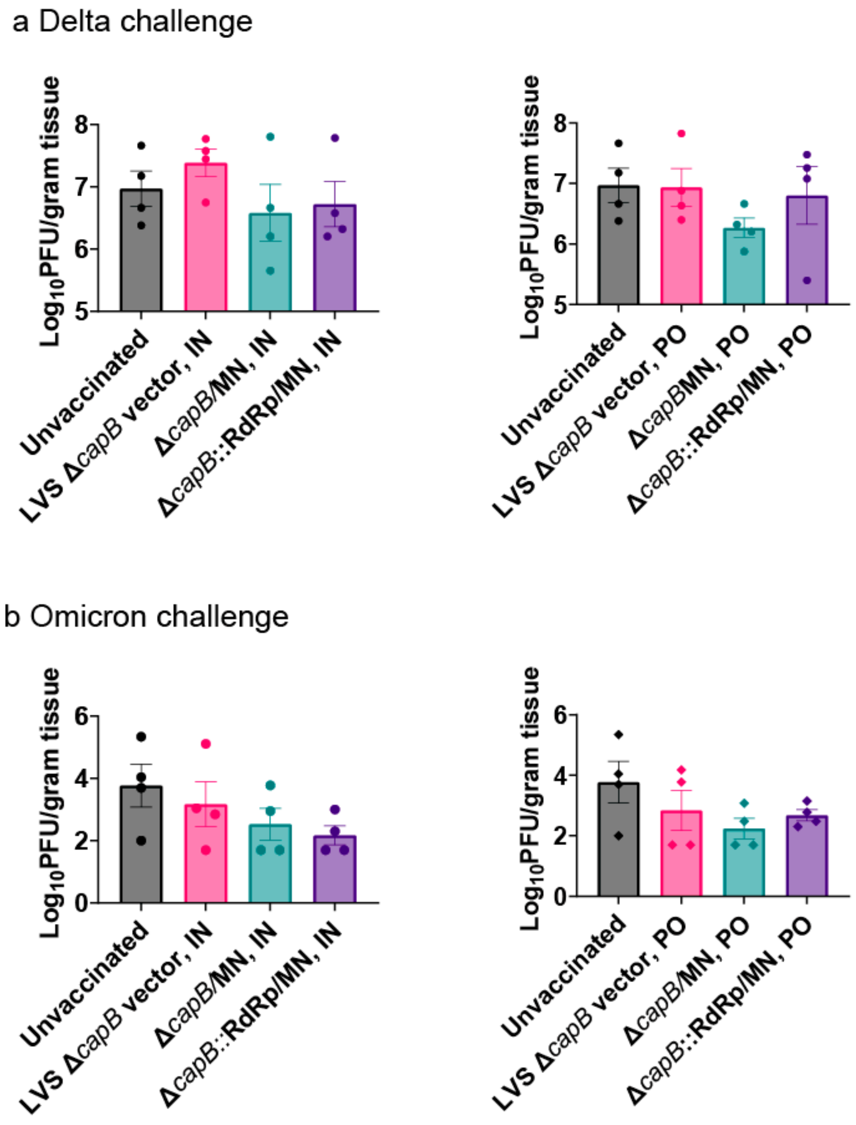

Supplementary Figure S2. Viral titers in the turbinates of hamsters immunized and challenged with SARS-CoV-2 Delta and Omicron strain. a. Turbinate viral titers after Delta variant challenge. Golden Syrian hamsters (8/group, half male, half female) were immunized three times, 3 weeks apart at Week 0 and 3, IN (a) with 2 x 10^6 CFU or PO (b) with 1 x 10^9 CFU rLVS Δ*capB*/MN or rLVS Δ*capB*::RdRp/MN vaccine candidates. Unvaccinated hamsters and hamsters immunized with equivalent IN or PO doses of the LVS Δ*capB* vector served as

controls. At Week 10, all the hamsters were challenged IN with 1.1 x 10^4 pfu of SARS-CoV-2 Delta variant (B.1.617.2-AY.1) and monitored closely for clinical signs of infection including weight loss. At Day 3 post-challenge, 4 animals (2M, 2F) in each group were euthanized and their turbinate tissues collected and assayed for virus load. Values are mean ± SEM and analyzed using one-way analysis of variance model. No significant differences between groups were found. **b**. Turbinate viral titers after Omicron variant challenge. Golden Syrian hamsters (8/group, half male, half female) were immunized twice, at Week 0 and 3, IN **(a)** with 2 x 10^6 CFU or PO **(b)** with 1 x 10^9 CFU of rLVS Δ*capB*/MN or rLVS Δ*capB*::RdRp/MN vaccine candidates. Unvaccinated hamsters and hamsters immunized with equivalent IN or PO doses of the LVS Δ*capB* vector served as controls. Seven weeks after the last immunization (Week 10), all the hamsters were challenged IN with 8.9 x 10^3 pfu Omicron Variant (BA.5) and monitored closely for clinical signs of infection including weight loss. Half of the hamsters (n=4/group) were euthanized at 3 days post-challenge and turbinate tissues were collected and assayed for viral load. Values are mean ± SEM and analyzed using one-way analysis of variance model. No significant difference between groups was found.

Supplementary Figure S3

a. Chemiluminescent image of Supplementary Figure S1 panel b

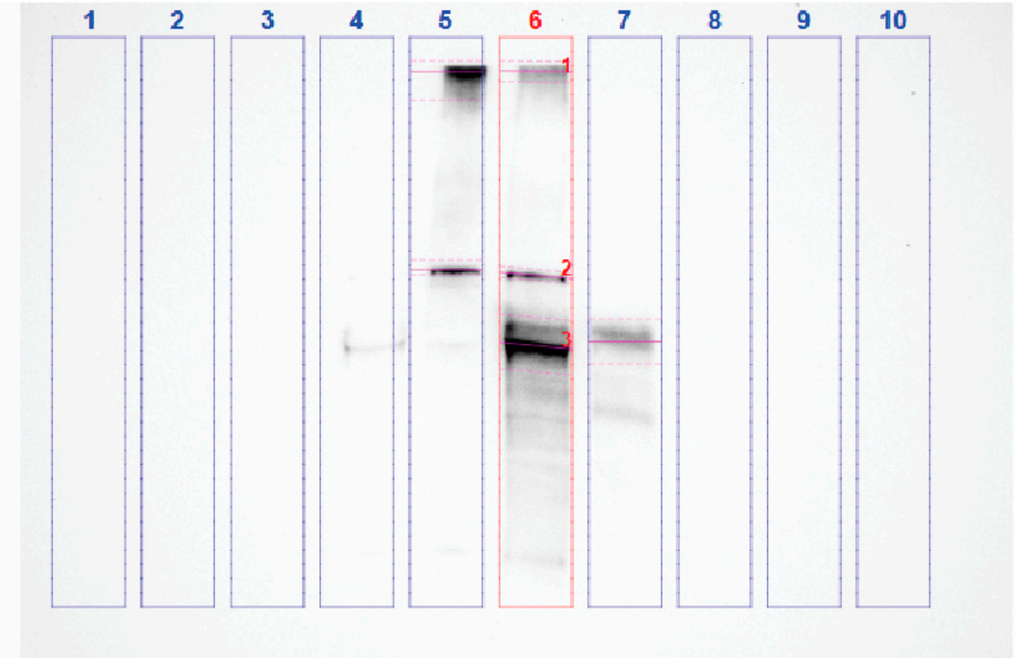

b. Densitometry readings of major protein bands

Lane	Band No.	Mol. Wt. (KDa)	Relative Front	Adj. Volume (Int)	Volume (Int)	Abs. Quant.	Rel. Quant.	Band %	Lane %
**5**	1	N/A	0.061	2.73E+07	4.69E+07	N/A	N/A	76.73	68.40
**5**	2	N/A	0.408	8.27E+06	1.67E+07	N/A	N/A	23.27	20.75
**6**	1	N/A	0.060	8.84E+06	1.92E+07	N/A	N/A	17.37	14.92
**6**	2	N/A	0.418	7.51E+06	1.37E+07	N/A	N/A	14.75	12.67
**6**	3	N/A	0.541	3.46E+07	1.03E+08	N/A	N/A	67.88	58.29
**7**	1	N/A	0.534	8.87E+05	3.75E+07	N/A	N/A	100.00	66.60

Supplementary Figure S3. Densitometry readings of each band in Supplementary Figure S1b using Image Lab software (Version 6.1.0. Build 7, Bio-Rad Laboratory). Lane 5 corresponds to lane 4 of Supplementary Figure S1b; lane 6 corresponds to lane 5 of Supplementary Figure S1b; and lane 7 corresponds to lane 6 of Supplementary Figure S1b.

Supplementary Figure S4

a. Chemiluminescent image of Supplementary Figure S1 panel c

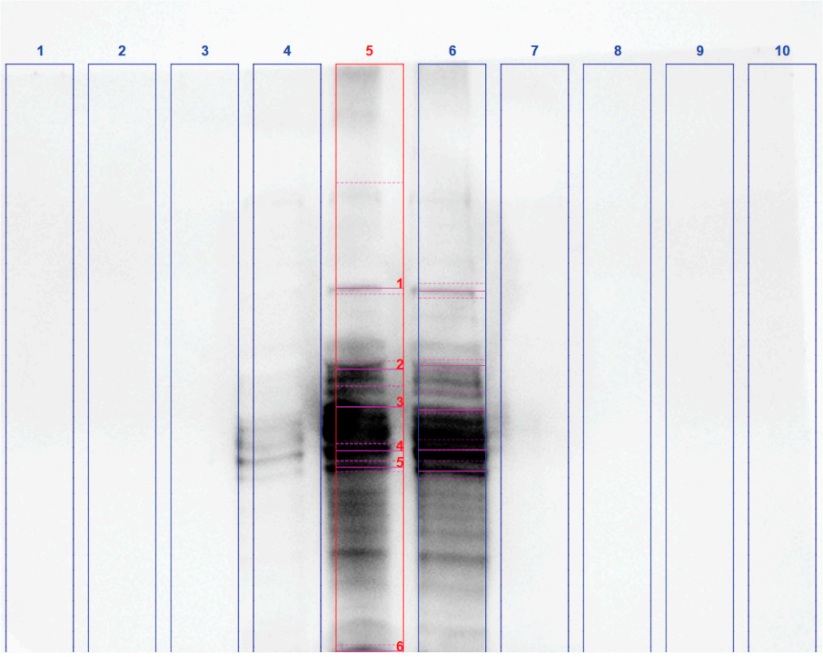

b. Densitometry readings of major protein bands

Lane	Band No.	Mol. Wt. (KDa)	Relative Front	Adj. Volume (Int)	Volume (Int)	Abs. Quant.	Rel. Quant.	Band %	Lane %
**5**	1	N/A	0.365	2.17E+06	6.50E+07	N/A	N/A	9.76	7.31
**5**	2	N/A	0.497	2.32E+06	4.51E+07	N/A	N/A	10.44	7.82
**5**	3	N/A	0.559	2.61E+06	1.30E+08	N/A	N/A	11.72	8.77
**5**	4	N/A	0.630	2.93E+06	3.88E+07	N/A	N/A	13.18	9.87
**5**	5	N/A	0.657	2.07E+06	2.24E+07	N/A	N/A	9.29	6.96
**5**	6	N/A	0.957	1.01E+07	2.60E+07	N/A	N/A	45.60	34.14
**6**	1	N/A	0.370	2.03E+06	1.18E+07	N/A	N/A	13.24	6.23
**6**	2	N/A	0.492	2.88E+05	1.35E+07	N/A	N/A	1.88	0.88
**6**	3	N/A	0.564	2.42E+06	3.58E+07	N/A	N/A	15.82	7.44
**6**	4	N/A	0.629	8.91E+05	2.81E+07	N/A	N/A	5.82	2.74
**6**	5	N/A	0.664	3.29E+06	4.15E+07	N/A	N/A	21.50	10.11
**6**	6	N/A	0.971	6.38E+06	1.50E+07	N/A	N/A	41.73	19.62

Supplementary Figure S4. Densitometry readings of each band in Supplementary Figure S1c using Image Lab software (Version 6.1.0. Build 7, Bio-Rad Laboratory). Lane 5 corresponds to lane 4 in Supplementary Figure S1c and lane 6 corresponds to lane 5 in Supplementary Figure S1c.

Supplementary Tables

Supplementary Table S1. Weight change post Delta variant challenge in IN-immunized hamsters

Days post-challenge	Unvaccinated	LVS Δ*capB* vector IN	rLVS Δ*capB/*MN IN	rLVS Δ*capB::RdRp*/MN IN
**0**	0.00	0.00	0.00	0.00
**1**	-3.20	-2.67	-3.37	-3.81
**2**	-4.83	-4.86	-4.90	-5.21
**3**	-5.74	-5.41	-3.63	-4.11
**4**	-7.75	-5.47	-5.00	-5.58
**5**	-8.75	-6.13	-4.57	-5.58
**6**	-9.70	-6.17	-4.50	-5.08
**7**	-8.20	-4.93	-4.20	-3.98
**% Max weight loss**	**-9.70**	**-6.17**	**-5.00**	**-5.58**

Supplementary Table S2. Weight change post Delta variant challenge in PO-immunized hamsters

Days post-challenge	Unvaccinated	LVS Δ*capB* vector PO	rLVS Δ*capB/*MN PO	rLVS Δ*capB::RdRp*/MN PO
**0**	0.00	0.00	0.00	0.00
**1**	-3.20	-3.07	-5.36	-4.30
**2**	-4.83	-5.64	-6.59	-5.97
**3**	-5.74	-7.69	-6.41	-5.77
**4**	-7.75	-8.57	-7.10	-5.37
**5**	-8.75	-9.37	-8.43	-5.80
**6**	-9.70	-9.97	-8.10	-5.00
**7**	-8.20	-10.07	-6.70	-4.70
**% Max weight loss**	**-9.70**	**-10.07**	**-8.43**	**-5.97**

Supplementary Table S3. Lung histopathological scores post Delta variant challenge

Group	Animal #	Tissue*	Overall lesion extent	Bron-chitis	Alveolitis	Pneumocyte hyperplasia	Vasculitis	Interstitial inflamma-tion	Total lobe score	Total lung score
**Unvaccinated infected**	**3**	R	1	1	2	2	1	2	9	19
		L	1	3	2	2	0	2	10	
	**4**	R	3	2	4	4	1	4	18	35
		L	2	3	3	4	2	3	17	
	**7**	R	4	3	5	5	4	4	25	46
		L	3	4	4	4	2	4	21	
	**8**	R	4	4	5	5	3	5	26	47
		L	3	3	4	4	3	4	21	
**LVS Δ*capB* Vector, IN**	**3**	R	2	0	3	3	2	3	13	29
		L	2	3	3	3	2	3	16	
	**4**	R	3	4	4	4	2	4	21	42
		L	3	3	4	4	3	4	21	
	**8**	R	2	2	4	4	2	4	18	37
		L	3	3	4	4	1	4	19	
**rLVS Δ*capB*/ MN, IN**	**4**	R	1	2	1	1	2	2	9	31
		L	3	3	4	4	4	4	22	
	**7**	R	3	2	4	4	4	4	21	43
		L	3	3	4	4	4	4	22	
	**8**	R	3	2	4	4	4	4	21	43
		L	3	3	4	4	4	4	22	
**rLVS Δ*capB*:: RdRp/MN, IN**	**3**	R	2	2	2	2	4	4	16	30
		L	2	3	1	0	4	4	14	
	**4**	R	0	0	0	0	0	0	0	12
		L	2	3	0	0	4	3	12	
	**7**	R	2	2	2	2	3	2	13	23
		L	2	3	0	0	3	2	10	
	**8**	R	3	2	4	4	3	4	20	40
		L	3	3	4	4	2	4	20	
**LVS Δ*capB* Vector, PO**	**3**	R	4	2	5	5	2	4	22	42
		L	3	3	4	4	2	4	20	
	**4**	R	3	2	4	4	2	4	19	39
		L	3	3	4	4	2	4	20	
	**8**	R	3	1	4	4	3	3	18	37
		L	3	3	4	4	1	4	19	
**rLVS Δ*capB*/ MN, PO**	**4**	R	2	1	2	2	4	4	15	34
		L	2	3	3	3	4	4	19	
	**7**	R	3	1	4	4	3	4	19	37
		L	2	3	3	4	3	3	18	
	**8**	R	4	3	5	5	3	5	25	46
		L	3	3	4	4	3	4	21	
**rLVSΔ*capB*:: RdRp/MN, PO**	**3**	R	2	0	2	2	0	2	8	28
		L	3	2	4	4	3	4	20	
	**4**	R	1	0	0	0	2	1	4	8
		L	1	1	0	0	0	2	4	
	**8**	R	3	2	4	4	2	3	18	36
		L	3	3	3	4	2	3	18	

* R: cranial; L, caudal.

Supplementary Table S4. Weight change post Omicron variant challenge in IN-immunized hamsters

Days post-challenge	Unvaccinated	LVS Δ*capB* Vector IN	rLVS Δ*capB/*MN IN	rLVS Δ*capB::RdRp*/MN IN
**0**	0.00	0.00	0.00	0.00
**1**	-1.64	-1.80	-2.29	-2.86
**2**	-3.59	-3.66	-4.08	-4.77
**3**	-4.50	-5.24	-3.74	-4.50
**4**	-3.85	-7.10	-2.25	-6.73
**5**	-4.08	-7.47	-1.00	-6.57
**6**	-3.95	-6.17	-0.78	-5.63
**7**	-3.40	-5.43	-0.08	-5.00
**% Max weight loss**	**-4.50**	**-7.47**	**-4.08**	**-6.73**

Supplementary Table S5. Weight change post Omicron variant challenge in PO-immunized hamsters

Days post-challenge	Unvaccinated	LVS Δ*capB* Vector PO	rLVS Δ*capB/*MN PO	rLVS Δ*capB::RdRp*/MN PO
**0**	0.00	0.00	0.00	0.00
**1**	-1.64	-0.43	-1.40	-1.13
**2**	-3.59	-2.39	-3.56	-2.93
**3**	-4.50	-3.39	-3.55	-1.81
**4**	-3.85	-5.28	-1.58	-0.95
**5**	-4.08	-5.30	-1.05	-0.78
**6**	-3.95	-5.35	+0.83	+0.03
**7**	-3.40	-4.20	+3.43	+0.18
**% Max weight loss**	**-4.50**	**-5.35**	**-3.56**	**-2.93**

Supplementary Table S6. Lung histopathological scores post Omicron variant challenge

Treatment	Animal #	Tissue	Overall lesion extent	Bron-chitis	Alveo-litis	Pneumocyte hyperplasia	Vasculitis	Interstitial inflammation	Total lobe score	Total lung score
**Unvaccinated Infected**	5	R	3	2	4	4	2	4	19	36
		L	3	2	4	4	1	3	17	
	6	R	1	1	2	2	1	1	8	25
		L	3	2	4	4	1	3	17	
	7	R	1	0	0	1	0	2	4	10
		L	1	0	1	2	0	2	6	
	8	R	1	0	2	2	2	2	9	20
		L	1	2	2	2	2	2	11	
**LVS Δ*capB* Vector, IN**	6	R	1	0	1	1	0	2	5	14
		L	1	0	2	3	0	3	9	
	7	R	1	0	2	3	0	3	9	27
		L	3	2	4	4	1	4	18	
	8	R	3	2	4	4	1	4	18	36
		L	3	2	4	4	1	4	18	
**rLVS Δ*capB*/ MN, IN**	5	R	0	0	0	0	0	0	0	0
		L	0	0	0	0	0	0	0	
	6	R	0	0	0	0	0	0	0	2
		L	1	0	0	0	0	1	2	
	7	R	1	0	0	0	4	2	7	13
		L	1	0	0	0	2	3	6	
	8	R	1	0	0	0	2	2	5	10
		L	1	0	0	0	2	2	5	
**rLVS Δ*capB*:: RdRp/MN, IN**	6	R	2	1	0	0	2	3	8	17
		L	2	1	0	0	3	3	9	
	7	R	3	0	3	3	0	4	13	29
		L	3	0	4	4	1	4	16	
	8	R	2	0	2	2	1	2	9	16
		L	1	0	2	2	0	2	7	
**LVS Δ*capB* Vector, PO**	5	R	3	1	4	4	1	4	17	33
		L	3	1	4	4	1	3	16	
	6	R	3	1	4	4	2	4	18	41
		L	4	3	5	5	1	5	23	
	7	R	2	0	2	3	0	2	9	20
		L	2	0	2	3	1	3	11	
	8	R	2	0	3	3	2	4	14	34
		L	3	2	4	4	3	4	20	
**LVS Δ*capB*/ MN, PO**	5	R	1	0	1	1	1	1	5	9
		L	1	0	1	1	0	1	4	
	6	R	0	0	0	0	0	0	0	0
		L	0	0	0	0	0	0	0	
	7	R	2	1	3	3	1	3	13	30
		L	3	0	4	4	2	4	17	
	8	R	0	0	0	0	0	0	0	5
		L	1	0	0	0	2	2	5	
**LVS Δ*capB*:: RdRp/MN, PO**	5	R	1	0	0	0	1	1	3	3
		L	0	0	0	0	0	0	0	
	6	R	1	0	0	0	2	2	5	12
		L	1	1	0	0	3	2	7	
	7	R	1	0	0	0	2	2	5	17
		L	2	1	2	1	3	3	12	
	8	R	2	0	0	0	4	3	9	18
		L	2	1	0	0	4	2	9	

* R: cranial; L, caudal.

Supplementary Table S7 – Amino acid differences between the M, N, and RdRp of the early SARS-CoV-2 isolates and the late Delta and Omicron variants

Protein	Variant	GenBank Locus	DBSOURCE Accession	Total Amino acid In the protein	Number of Mutations	% difference
**Membrane**	Wuhan-hu1	QHD43419.1	MN908947.3	222		
	WA1-2020	QKJ89360.1	MT576556.1	222	0	0.00
	Delta	UAL04650.1	OK091006.1	222	1	0.45
	Omicron	WAL91236.1	OP984772.1	222	3	1.35
**Nucleocapsid**	Wuhan-hu1	QHD43423.2	MN908947.3	419		
	WA1-2020	QKJ89365.1	MT576556.1	419	0	0.00
	Delta	UAL04655.1	OK091006.1	419	4	0.95
	Omicron	WAL91241.1	OP984772.1	416	7	1.67
**RdRp**	Wuhan-hu1	YP_009725307.1	MN908947.3	932		
	WA1-2020	QKJ89355.1	MT576556.1	932	0	0.00
	Delta	UAL04645.1	OK091006.1	928	6	0.64
	Omicron	WAL91231.1	OP984772.1	928	5	0.54
**Spike**	Wuhan-hu1	QHD43416	MN908947.3	1273		
	WA1-2020	QKJ89357.1	MT576556.1	1273	0	0.00
	Delta	UAL04647.1	OK091006.1	1273	11	0.86
	Omicron	WAL91233.1	OP984772.1	1268	35	2.75
